# Word decoding development in incremental phonics instruction in a transparent orthography

**DOI:** 10.1007/s11145-017-9735-3

**Published:** 2017-03-07

**Authors:** Moniek M. H. Schaars, Eliane Segers, Ludo Verhoeven

**Affiliations:** Behavioural Science Institute, Radboud University, Montessorilaan 3, 6525 HR Nijmegen, The Netherlands

**Keywords:** Decoding, Phonics instruction, Early literacy, Phonological awareness, Longitudinal

## Abstract

The present longitudinal study aimed to investigate the development of word decoding skills during incremental phonics instruction in Dutch as a transparent orthography. A representative sample of 973 Dutch children in the first grade (*M*_*age*_ = 6;1, *SD* = 0;5) was exposed to incremental subsets of Dutch grapheme–phoneme correspondences during 6 consecutive blocks of 3 weeks of phonics instruction. Children’s accuracy and efficiency of curriculum embedded word decoding were assessed after each incremental block, followed by a standardized word decoding measurement. Precursor measures of rapid naming, short-term memory, vocabulary, phonological awareness, and letter knowledge were assessed by the end of kindergarten and subsequently related to the word decoding efficiency in the first grade. The results showed that from the very beginning, children attained ceiling levels of decoding accuracy, whereas their efficiency scores increased despite the incremental character of the consecutive decoding assessments embedded in the curriculum. Structural equation modelling demonstrated high stability of the individual differences assessed by word decoding efficiency during phonics instruction during the first 5 months of the first grade. Curriculum embedded word decoding was highly related to standardized word decoding after phonics instruction was completed. Finally, early literacy and lexical retrieval, and to a lesser extent verbal and visual short term memory, predicted the first fundamental processes of mastering word decoding skills.

## Introduction

Emergent literacy programs have become an essential part of preschool and kindergarten programs and policies nowadays. By storybook reading and interactive language games, these programs playfully focus on phonological awareness, grapheme-to-phoneme knowledge, and vocabulary. As a result, children make substantial progress in these domains (Piasta & Wagner, 2010; Whitehurst & Lonigan, 2001; Yaden, Rowe, & MacGillivray, 1999), and such affordances predict children’s word decoding development in subsequent years (Bus & Van Ijzendoorn, 1999). Although encouraging, these emergent literacy programs do not reduce the importance of systematic phonics instruction in learning to read in the first grade (Ehri, Nunes, Stahl, & Willows, 2001). In systematic phonics instruction, prespecified sets of phonic elements, such as grapheme–phoneme correspondences, are incrementally being taught and simultaneously applied in reading words and text. Therefore, phonics instruction helps children to develop reading skills. Only very few studies have documented the steps that children make to progress from pre-alphabetic phases to actual alphabetic reading. Thus, the ways in which children internalize an increasing stock of essential grapheme–phoneme correspondence and blend rules remain unclear. Additionally, the relationships between these initial steps in reading development and the cognitive and linguistic abilities that children have attained before the start of formal reading instruction have not been identified. Therefore, in the present study, we examined the development of word decoding skills in incremental phonics instruction in Dutch as a transparent orthography.

Learning to read typically evolves in different phases along a continuum (Ehri, 2005; Ehri & McCornick, 1998), starting with the transition from a nonreading pre-alphabetic phase in kindergarten, to a partial alphabetic phase, and finally to a full alphabetic phase of reading during first grade. During these initial phases, children gradually learn how graphemes systematically correspond to phonemes and how they can blend the phonemes to form words. Reading words by systematically mapping and blending the phonological elements within words is called phonological recoding. Phonological recoding has been proposed to be a fundamental aspect of successful reading development (Share, 1995). It allows children to translate unfamiliar words from symbols into sounds without ‘external’ feedback from a teacher. Hence, this solid baseline of phonological recoding functions as a self-teaching mechanism for further word decoding development (Share, 1995, 1999, 2004). Through repetitive word decoding, words or word parts will be stored in a so called orthographic lexicon (Coltheart, Rastle, Perry, Langdon, & Ziegler, 2001). With growing reading experience, words in the orthographic lexicon become better specified (Perfetti, 1992), which enables children to deploy orthographic information directly from the memory instead of actively converting and combining all graphemes into phonemes. This makes word decoding faster, more efficient, and it provides children with additional equipment while decoding words which include unfamiliar graphemes (Gilbert, Compton, & Kearns, 2011).

Systematic phonics-based instruction methods are based on the assumption of incrementally building a solid baseline of alphabetic knowledge in order to further support the building of an orthographic lexicon through the self-teaching mechanism. Explicit incremental instruction provides children with a systematic guidance through this phase of mastering the alphabetic principle. Simultaneously, the development of the self-teaching mechanism of word decoding is optimally triggered. During incremental phonics instruction, a small set of grapheme–phoneme correspondences is first presented to the children who practice them by reading words and short sentences comprising trained graphemes. After an intensive training with this first set of graphemes, subsequent sets of new graphemes are incrementally added to the baseline set. Every time a set of new graphemes is added, the full set of graphemes is repeatedly practiced in words and sentences to give children the opportunity to apply and consolidate all grapheme–phoneme correspondence and blend rules that have been acquired (see Ellis & Ralph, 2000). This controlled environment of learning to read provides an opportunity for children to practice conversion rules and blend skills without being bothered by unknown graphemes and orthographic units that they have not been taught yet. Evidence suggests that systematic phonics instruction is highly successful in teaching children word decoding (e.g., Ryder, Tunmer, & Greaney, 2008). Ehri et al.’s (2001) meta-analysis of 38 studies clearly showed that systematic phonics instruction was more effective compared to nonsystematic phonics instruction or whole language instruction, even in the opaque English orthography. Furthermore, in a direct comparison, De Graaff, Bosman, Hasselman, and Verhoeven (2009) introduced either a systematic phonics approach (i.e., prespecified sets of phonics elements) or a nonsystematic phonics approach (i.e., arbitrary introducing of phonics elements) to children who had not received formal reading instruction yet. Although the grapheme–phoneme knowledge progressed similarly in both approaches, the phonemic awareness skills and the word decoding skills progressed more in the systematic approach. Powell, Plaut, and Funnell (2006) previously suggested this powerful combination of systematic training on grapheme-to-phoneme correspondences on the one hand, and incremental training on actual word reading on the other hand by simulating the children’s reading development using a statistical computer network. However, the fit of their highly controlled network to the children’s actual reading development was only partially successful. The partially successful fit emphasized the additional influence of more than just these two variables on the reading development of real children.

A prominent question that remains, concerns the stability of the development of word decoding skills during the first months of systematic phonics instruction in the first grade. Longitudinal reading studies in both transparent (e.g., Bast & Reitsma, 1998; Verhoeven & Van Leeuwe, 2009) and more opaque orthographies (e.g., Caravolas, Lervåg, Defior, Málkova, & Hulme, 2013; Juel, 1988; Simmons et al., 2008; Steacy, Kirby, Parrila, & Compton, 2014) evidenced high individual stability in reading development over time. However, reported stability in these studies typically refers to the development that was observed after, instead of during, the first months of reading instruction during which fundamental word decoding skills are mastered. The focus should be on the very beginning of reading development to better understand how children develop these fundamental word decoding skills. Only very few studies reported the development of reading skills during the months in which the grapheme–phoneme correspondences are being taught.

Spector (2005) evidenced instability of reading and reading related skills prior to stabilization in the first year of reading instruction for children learning to read in English. However, the repeated measures design was not fine-grained enough to capture the actual acquisition of early reading processes. Moreover, during the months covered in Spector’s study, most children had not fully mastered the alphabetic principle. Yet, reading was measured with tests that assumed full alphabetic knowledge, resulting in floor level scores. Measures of reading development should closely evolve with the developmental process to be able to capture the steps in early reading development. Additionally, this study was conducted among poor readers only, so no further knowledge of early reading acquisition in general was provided. Juul, Poulson, and Elbro (2014) assessed word reading with relatively short measurement intervals. In their study among 172 children, accuracy and speed of word decoding was assessed every second month during the first two years of formal reading instruction. The test material consisted of lists with a total of 24 CVC-structured words that were repeated at every test moment. Juul et al. evidenced ongoing progress in both reading accuracy and speed from the beginning. Compton (2000) documented the fine grained and early development of 75 first grade children learning to read in English and showed on average a linear growth of accuracy in curriculum based word decoding skill. However, in both studies, no details about decoding efficiency and the individual stability of decoding efficiency over time were provided. Furthermore, it is important to note that early reading development has almost uniquely been studied in the English orthography. In the opaque English orthography, reading is hampered by complex orthographic irregularities from the very beginning (see also Share, 2008). Due to the opaque orthography, only a sided view of the early steps in reading development was provided.

A second prominent question concerns the point when children’s cognitive and linguistic abilities in kindergarten become relevant in the development of word decoding skills. Cognitive and linguistic abilities, like rapid naming, short-term memory, vocabulary, and early literacy (i.e., phonemic awareness, grapheme–phoneme knowledge) are already developing prior to reading. The quality of these abilities prior to reading is assumed to be related to later reading development. It has been established that the individual differences in these precursors, measured prior to formal reading instruction, are substantial (e.g., Al Otaiba & Fuchs, 2002; Kirby, Desrochers, Roth, & Lai, 2008; Kirby et al., 2010; Landerl et al., 2013; Melby-Lervåg, Lyster, & Hulme, 2012; Moll et al., 2014; Nelson, Benner, & Gonzalez, 2003). In addition, individual differences in visual and verbal short-term memory have been shown previously (e.g., Bosse & Valdois, 2009; Van den Boer, De Jong, & Haentjens-van Meeteren, 2013). The influence of these core predictors is assumed to be more or less universal, as it has been shown in both transparent and more opaque orthographies (Caravolas et al., 2013; Vaessen et al., 2010). However, relative contributions of the precursors might vary between orthographies (Caravolas et al., 2013; Ziegler & Goswami, 2005, 2006) and between phases of reading development (e.g., De Jong & Van der Leij, 1999; Georgiou, Papadopoulos, & Kaizer, 2014; Papadimitriou & Vlachos, 2014; Wagner et al., 1997). The existing research has so far mostly focused on the predictive power of the precursors in word decoding skills by the end of first grade or beyond (but see Compton, 2000). Little attempt has been made to explore how such precursors influence the development of word decoding skills in incremental phonics instruction in a transparent orthography.

To sum up, in the research so far, the development of word decoding skills during systematic phonics instruction has received only scant attention. A full understanding of underlying processes during the development of word decoding skills remains incomplete, since most longitudinal designs started only after the fundamental skills had been mastered. Furthermore, they largely lack fine-grained short interval measurements, and they assess reading development mostly only in terms of accuracy, disregarding development of word decoding efficiency. Consequently, it is by no means clear how accurate and efficient beginning readers are during the period during which systematic sets of grapheme–phoneme correspondences are being taught and practiced. The stability in individual differences of reading development has previously been evidenced, but only after the first period of initial phonics instruction. No clear case was provided during, instead of after, the development of fundamental word decoding skills in early phonics instruction. It is also not clear how precursors assessed by the end of kindergarten relate to the development of word decoding skills during phonics instruction in a transparent orthography.

The goal of the current study was to examine the development of word decoding skills in incremental phonics instruction in a transparent orthography. For this purpose, we systematically measured word decoding accuracy and efficiency (defined as the amount of correctly read words per minute) after every incremental step that Dutch children make during initial phases of learning to read. Dutch can be seen as a relatively transparent orthography (Seymour, Aro, & Erskine, 2003), which implies that words can generally be decoded on the basis of grapheme–phoneme correspondence rules. Most studies on reading development have been conducted in the opaque English orthography (Share, 2008). In English, children already have to acquire some knowledge of complex orthographic irregularities (e.g., homophones, homographs) when they first start learning to read. Therefore, transparent and opaque aspects of orthographic learning become intertwined in beginning reading. Dutch gives the unique opportunity to study the development of mastering the alphabetic principle during the first months of formal reading instruction, as word decoding is not hampered by complex orthographic irregularities. A second advantage of studying early reading development in the Netherlands is that most schools use one and the same reading curriculum in which learning to read follows a uniform systematic procedure of implementing incremental sets of grapheme–phoneme correspondence rules along with word decoding and simple text comprehension practice. The combination of a relatively transparent orthography and a highly systematic phonics-based reading method sets the stage to study the more general and universal aspects of early word decoding development without interference from distracting orthographic irregularities.

The current study monitors the development of word decoding by a predefined systematic phonics-based instruction in Dutch. An attempt was made to find an answer to the following questions:

- How accurate and efficient are incremental curriculum embedded word decoding skills of Dutch children during phonics instruction, and how stable is its development over time?
- To what extent are curriculum embedded measurements of word decoding skills related to standardized measurement of word decoding after initial phonics instruction is completed?
- To what extent can the development of incrementally built-up word decoding skills be predicted from children’s rapid naming, short term memory, vocabulary, phonological awareness, and letter knowledge in kindergarten?

With respect to the first question, it was hypothesized that Dutch children would be highly accurate in word decoding from the very beginning. Full acquisition of word decoding skills was expected to be largely a matter of growing efficiency, as indicated by increasing numbers of words being read accurately per minute. We also expected high stability in the development of incrementally built-up word decoding skills over time. This implies that word decoding levels would be highly predictable from earlier word decoding performances across the 5 months of initial phonics instruction. Furthermore, it was hypothesized that the development of word decoding skills, as measured by curriculum embedded measurements during phonics instruction, would be highly related to standardized word decoding measurements after the first months of formal instruction.

With reference to the second question, it was hypothesized that the development of word decoding skills could primarily be explained by children’s levels of phonemic awareness skills and grapheme–phoneme knowledge, and to a lesser extent also by their levels of rapid naming, short-term memory, and vocabulary as measured before the start of phonics instruction. In addition to phonemic awareness, we expected rapid naming to be of high predictive value immediately, since we hypothesized that in this Dutch sample, word decoding would be a matter of increasing efficiency from the very beginning of learning to read. Furthermore, we hypothesized that short term memory skills might become more important later in development when words are longer and decoding tasks become more demanding. In addition, it was hypothesized that vocabulary would be of less importance during the development of word decoding skills, since the words in the beginning of learning to read by phonics instruction in the relatively transparent Dutch orthography can be decoded without explicit orthographic or semantic knowledge.

## Method

### Participants

In the Netherlands, children start kindergarten the day they reach the age of 4. Subsequently, they generally attend at least two years of kindergarten. In kindergarten, children are playfully being introduced to some grapheme–phoneme correspondences, and attention is being given to the development of phonological awareness. Formal reading instruction starts in Grade 1, at about the age of 6.

We invited schools via a general mailing to participate in a large longitudinal cohort study on reading development following the kindergarten. All invited schools were using the same systematic and predefined incremental phonics method *Veilig Leren Lezen* (“Learning to Read Safely”; Mommers et al., 2003) as a reading method in Grade 1. This method is being used by 87% of all schools in the Netherlands. A sample of 37 primary schools throughout the Netherlands, located in both rural and urban areas, participated in the longitudinal study. Children who were expected to stay in kindergarten for an additional year were excluded from the participation in this study beforehand. Passive informed consent was obtained from the parents of 1006 children. For the current study, these children were monitored from the end of kindergarten halfway through Grade 1. Children who missed more than three consecutive assessments on the monthly curriculum-embedded word decoding tasks in Grade 1 (3.3% of participants) were excluded from the analyses. These structural exclusions were mostly due to movements or transfer to other schools or to substantial absence due to lasting illness during Grade 1. The final sample consisted of 973 Dutch children (505 boys; 468 girls).

At the start of the longitudinal study, during the assessment at the end of kindergarten, the mean age of the children was 6;1 years (*SD* = 0;5). All participating children spoke Dutch at school. Sixteen percent of the children were second language learners and spoke another language at home, representing the multicultural nature of the population in the Netherlands. The socioeconomic status of the children, as indicated by the educational level of their main caregiver (response rate was 77% of the sample), roughly displayed the distribution of educational level in the Netherlands (Centraal Bureau voor de Statistiek [Statistics Netherlands], 2013).

### Materials

#### Word decoding in Grade 1

*Incremental phonics approach* In Grade 1, all participating schools used the same systematic incremental phonics-based reading instruction method. This method consists of extensive manuals and schedules, and the lessons and materials are well defined to ensure consistency in education between schools. The same lessons are provided to all children, with the possibility to make small adjustments to individual exercises to accommodate children’s levels. These adjustments are recommended to the teacher by curriculum embedded teacher software based on the logged test scores of individuals. During 5 months of formal reading instruction, all 34 graphemes that have to be learned in Dutch reading are covered in each classroom.

**Fig. 1:**
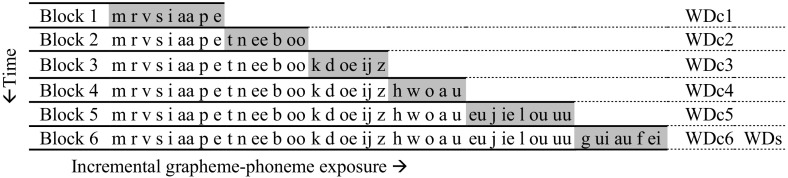
Six consecutive blocks of incremental introduction of grapheme–phoneme correspondences with an assessment of curriculum embedded Word Decoding efficiency (WDc) after each training block and a standardized Word Decoding efficiency assessment (WDs) after six blocks. Newly introduced graphemes per training block are marked in *grey*

The instruction method for daily reading instruction in Grade 1 is characterized by systematic incremental introduction and practise of new grapheme–phoneme correspondences. Instruction during the first 5 months consists of six successive blocks of three-to-four weeks (see Fig. 1). In the first block, a set of eight graphemes is repeatedly presented to the children and practiced in mono-syllabic CVC-words and small sentence contexts. In every subsequent training block, a predefined set of five or six new graphemes is added to the baseline training set until 34 Dutch grapheme–phoneme correspondences are included after six blocks (i.e., all graphemes used in Dutch except from *c*, *q*, *y*, *x*). The curriculum increments follow phonological pronunciation rules. For example, children start with learning the m, r, and s, as these graphemes can be prolonged when pronouncing them, in contrast to plosives. Every time new graphemes are added to the training set, the total set of graphemes is practiced in word and sentence contexts.

*Curriculum embedded word decoding* We monitored the progress of word decoding with curriculum embedded word decoding tasks. Curriculum Embedded Measurements (CEMs) assess the mastery levels of skills that have been taught explicitly, evolving in alignment with instruction (Oslund et al., 2015). Curriculum embedded assessments are suitable methods for obtaining meaningful information from the very first steps in reading development while standardized measurements would be too rough and general to identify individual differences (floor level results). Such subsequent progress monitoring assessments are typically nonequivalent. The content of each consecutive measurement has been determined in parallel with the incrementally growing set of trained graphemes to measure the mastery level up till that moment. The first card, used after four weeks of formal instruction, consists of 30 monosyllabic CV/CVC/VC-structured words. The words comprise the eight graphemes that have been trained during the first training block (10 words occur twice on this card because too few graphemes have been acquired yet to form 30 words). Each subsequent curriculum embedded word decoding card comprises 40 monosyllabic CV/CVC/VC-structured words consisting of incrementally trained graphemes. The words on the cards are presented in columns of 10 words, all with consistent grapheme–phoneme correspondences, and all are high frequent words with meanings that are familiar to the children. The child was asked to read out aloud the words on a card as accurately and quickly as possible for 1 min. If reading one word took more than 5 s, the correct answer was given and the child was asked to go on with the next word on the list. Not decoded words and incorrectly decoded words were scored as ‘inaccurately read’. The sum of the accurately read words within 1 min (i.e., the efficiency score) was the score on the task.

*Standardized word decoding* The national standardized word decoding test for halfway Grade 1 (*Drie*-*minutentoets*; “Three-minutetest”; Krom, Jongen, Verhelst, Kamphuis, & Kleintjes, 2010) consists of two cards, one card with five columns of 30 high-frequency monosyllabic CV/CVC/VC-structured words and a second card with five columns of 30 high-frequency monosyllabic words, including words with consonant clusters in initial or final positions (CC or CCC). The child was asked to read aloud the words on each card as accurately and quickly as possible for 1 min per card. For each card, the efficiency score was logged. A combination score of cards 1 and 2 halfway through Grade 1 was reported reliable, with a Cronbach’s alpha of .96 (Krom et al., 2010). High correlations between the two card scores were also found in the current study (r = .86). The combined score of these two cards was used as an indicator of standardized word decoding efficiency halfway through Grade 1.

#### Cognitive and linguistic precursors

The kindergarten test consists of seven tasks on cognitive and linguistic precursors, and it was designed specifically for the purpose of the present study. Except from the grapheme–phoneme knowledge task, practice items preceded every actual task. On each task, the number of correct responses was the score.

*Early literacy* In the domain of early literacy, three tasks were administered, as described below.

*Phoneme isolation* Phoneme isolation skill was measured with a task in which the child was asked to sound out the first phoneme of 10 orally offered monosyllabic CVC-structured words. After five consecutive incorrect responses, the task was terminated to avoid further frustration for the child. The reliability of the task was good (Cronbach’s alpha = .83).

*Word segmentation* To assess word segmentation skills, the child was asked to serially pronounce each phoneme of an orally offered word. This task comprises 10 words with increasing difficulty (from CVC-structured words to CCVC- or CVCC to CCCVC- or CVCCC-structured words). The task was terminated after five consecutive incorrect responses. The reliability of the task was good (Cronbach’s alpha = .85).

*Grapheme*–*phoneme knowledge* To measure grapheme-to-phoneme conversion skills, the child was asked to sound out 34 graphemes used in Dutch. The graphemes *c*, *q*, *x*, and *y* were excluded from this task because these graphemes are infrequent in Dutch reading system and not yet introduced in the reading method in Grade 1. The graphemes were printed in lower case across three columns on a card. Arial (Monotype, Microsoft) font type of size 28 was chosen because of the clarity of a sans serif style and the similarity with the font used in the reading curriculum. In this task, only the grapheme sound not the grapheme’s name was considered correct. The reliability of the task was excellent (Cronbach’s alpha = .93).

Since we expected a high interrelationship among the three measures of early literacy, we conducted principal axis factor analysis with oblique rotation (Promax; Muthén & Muthén, 2007) on the three measures described above. The analysis revealed one component with high loadings (.83 to .84) that explained 70.09% of the variance. The Kaiser–Meyer–Olkin measure verified the adequacy of this analysis, KMO = .71 (middling; Hutcheson & Sofroniou, 1999). All analyses in the current study were conducted using this factor score of Early Literacy.

*Rapid naming* Rapid naming was measured with a lexical retrieval task of visually presented objects. The child was asked to name pictures from top to bottom as quickly and accurately as possible during one minute. The task consists of five repetitive pictures, all corresponding with one-syllable high frequent Dutch words (viz., *saw, pot, thumb, trousers, tent*). The five pictures are repeated at random positions in six columns of 22 objects each. The reliability of the task was excellent (Cronbach’s alpha = .95).

*Verbal short*-*term memory* To measure verbal short-term memory, a pseudoword repetition task was used. The research assistant orally introduced a pseudoword and the child was asked to accurately repeat the word. The task consists of 20 one-to-four syllable pseudowords in ascending order of length. After five consecutive incorrect responses, the task was terminated. The whole word had to be repeated correctly; stress differences and substitutions due to certain articulation errors in individuals were counted as correct. The reliability of the pseudoword repetition task was good (Cronbach’s alpha = .77).

*Visual short*-*term memory* To measure the sequential short-term memory of concrete visual information, the children were asked to remember the order of the series of visual presented figures (viz., *fish*, *cow*, *ship*, *chicken*, *sock*) that were presented in a booklet. A series was shown for 5 s by the research assistant. After 5 s, the research assistant closed the booklet and asked the child to put cards with the figures in the same order as had been presented in the booklet. The entire task consists of 15 series of figures, with the number of figures in a series increasing from two to five figures. The entire series had to be remembered to be considered correct. After three consecutive incorrect series, the task was terminated. The reliability of the visual short-term memory task was good (Cronbach’s alpha = .77).

*Vocabulary* The vocabulary task was developed to measure the active vocabulary of the child. Pictures in the task were extracted from the vocabulary task in the *Taaltoets Allochtone Kinderen* (“Language test Ethnic minority Children”; TAK; Verhoeven & Vermeer, 1986). Twenty-nine suitable and age appropriate picture-word combinations were selected based on the standard scores of the original task. Both nouns and verbs are included in the task. Twenty-nine black and white line pictures were shown to the child. Every picture was accompanied by a little phrase pronounced by the research assistant (e.g., ‘*The man is …*’). The child had to complete the phrase by naming the correct word (e.g., ‘*fishing*’). The task was terminated after five consecutive incorrect responses. The reliability was good (Cronbach’s alpha = .83).

### Procedure

At the end of kindergarten, an individual assessment of about thirty minutes was conducted to screen baseline precursors, which were assumed to be involved in reading development. The tasks in the test battery were administered by the first author and eight trained research assistants with Bachelor or Master’s degrees in Educational Science, Psychology, or Linguistics. The tasks were conducted in the same fixed order for all children. The research assistant orally provided instructions for all tasks. If necessary, instruction was repeated. During the practice items, some help and feedback was allowed, but during the actual tasks, no feedback on the correctness of item scores was provided to the children. There was a small pause between every task before the instruction for the next task started. All tasks were administered individually in a quiet room at school during regular school hours.

During the first 5 months of Grade 1, the data on word decoding ability were collected using a longitudinal design. As word reading develops very fast during the first year, the measures of reading development should evolve with that process to be able to identify the steps in acquisition (Ehri & McCormick, 1998). To capture each incremental step the children made, the children were individually assessed after each training block of 3-to-4 weeks (i.e., six times; see Fig. 1) on their ability to decode words that were constructed with the graphemes they had learned up until then. Assessments were conducted using curriculum embedded word decoding tasks of 1 min, carried out by certified classroom teachers of the participating schools (mostly the daily teachers of the children). The teachers were all instructed in the same way, and they were all experienced in how to conduct the assessments of the used reading method.

The treatment fidelity was ensured. Curriculum embedded word decoding was assessed in predefined fixed time paths. The online log systems showed that teachers first finished the curriculum defined instruction block before assessing the children with the curriculum embedded word decoding task, and assessments were conducted before the new instruction block started.

**Fig. 2:**
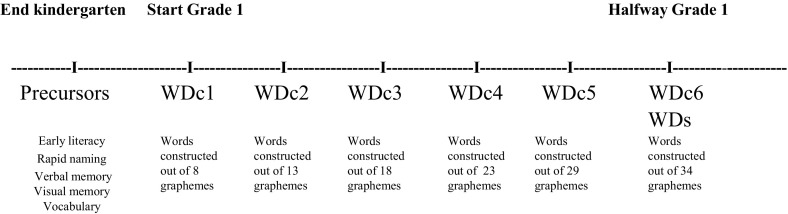
Timeline of assessments. First, the assessment of kindergarten cognitive and linguistic skills, followed by six consecutive curriculum embedded assessments of Word Decoding efficiency (WDc) after each training block, and a standardized Word Decoding efficiency assessment (WDs) after six blocks

After 5 months of formal reading education, a national standardized word decoding task was administered to determine the word decoding level independent of the reading curriculum that was used during the training phase. Following test guidelines, this task was assessed by teachers of the participating schools and was administered individually in a quiet room at school during regular school hours. See Fig. 2 for an overview of all measurement moments.

### Analytic approach

The number of words read correctly per minute (i.e., efficiency score) and the percent correct scores of actually read words (i.e., accuracy score) were computed and analysed for each consecutive curriculum embedded word decoding assessment (ANOVA RM). We conducted Sequential Equation path Modelling (SEM) in LISREL (Jöreskog & Sörbom, 1996) to examine the hypothesized stability of word decoding development in the first 5 months of instruction and to relate the curriculum embedded word decoding efficiency to a standardized measurement of word decoding efficiency after 5 months. A longitudinal design with time as a fixed factor was used, meaning that variables later in time were considered not to influence variables earlier in time. In addition to the consecutive autoregression (lag-1), we added lag-2 relations to the model. This additional influence on the forthcoming measurement moment thereafter strengthens the autoregression because the data pooled across two blocks is less noisy compared to the data from just the block before. Theoretically, it was assumed that these relations showed consolidation of learned connections. In the second model, precursors were added to the longitudinal model to measure their predictive value on the early word decoding development in Grade 1. Only influences that were significant at α < .05 were included in the models. After testing the hypothesized models, the Modification index and the associated Expected parameter changes were consulted in LISREL to ensure that no further plausible modifications were proposed within the context of our theoretically based hypotheses (see Little, 2013; Saris, Satorra, & Van der Veld, 2009).

The fit of the models was evaluated using Chi-square statistics (χ^2^). Because of the longitudinal character and the large sample size in the current study, the power to reject the model might be too high to use only Chi-square statistic as a decisive criterion (Jaccard & Wan, 1996; Little, 2013). Therefore, the Root Mean Square Error of Approximation (RMSEA; Little, 2013; Steiger & Lind, 1980) and the relative Chi-square (χ _rel_^2^), calculated as the ratio of the Chi-square with the degrees of freedom, were also evaluated. As a guideline for accepting the model, the RMSEA cut-off criterion was set at <.06 (Hu & Bentler, 1999; Tabachnik & Fidell, 2014), and the relative Chi-square should be lower than 3 (Carmines & McIver, 1981).

Prior to the analysis, we examined all variables for missing values. There were no missing values in the precursor tasks. Of the six curriculum embedded word decoding assessments and the standardized word decoding task, less than 3% of the single values were missing in total. Therefore, the dataset was suitable to estimate the parameters using the Full Information Maximum Likelihood (FIML) approach in LISREL (Enders, 2010; Little, 2013). To do so, we imported the raw data into LISREL and used an EM-procedure to obtain starting values for the FIML procedure. As a consequence, the LISREL output included global goodness of fit statistics and no additional relative fit indices.

## Results

Our first research question addressed the accuracy and efficiency of curriculum word decoding measurements during the first months of phonics instruction up to full alphabetic word decoding. Table 1 shows the means and standard deviations for the accuracy and efficiency scores of the six consecutive curriculum embedded Word Decoding tasks (WDc1–WDc6) together with the cognitive and linguistic precursor measures, and the score of the standardized Word Decoding test (WDs). Children achieved on average over 90% accuracy (*M* = 92.38%, *SD* = 10.78) in word decoding from the first measurement moment on (i.e., after 4 weeks of formal reading instruction). For word decoding efficiency, repeated measures analysis of variance (ANOVA RM) with measurement moment of Word Decoding efficiency (WDc) as independent variable showed a significant effect of measurement moment, *F* (5,4280) = 507.61, *p* < .001, *η* ^2^ = .37. This indicated that there was a significant increase of word decoding efficiency (despite the fact that the consecutive tasks increased in difficulty). Paired sample *t* tests showed that this growth was significant between every consecutive measurement moment, *p* < .001. The differences represented small to medium-sized effects, *d* = .22 − .63 (see Table 2). All further analyses on word decoding were performed using word decoding efficiency scores.

**Table 1:** Mean scores and standard deviations for precursor measures, measures of curriculum embedded word decoding and standardized word decoding

Measure	*M*_Accuracy_ (SD)	*M*_Efficiency_ (SD)
Precursor		
Phoneme isolation	8.24 (2.16)	
Word segmentation	4.37 (2.63)	
Grapheme–phoneme knowledge	18.71 (7.59)	
Rapid naming		39.57 (9.18)
Verbal short-term memory	14.82 (3.39)	
Visual short-term memory	8.27 (2.94)	
Vocabulary	13.47 (4.53)	
Curriculum embedded word decoding^a,b^		
WDc1	91.63 (13.20)	19.69 (14.76)
WDc2	92.39 (10.66)	22.77 (14.50)
WDc3	92.70 (11.57)	24.32 (17.01)
WDc4	92.61 (9.58)	27.32 (17.57)
WDc5	91.82 (10.88)	29.47 (19.98)
WDc6	93.14 (8.80)	34.49 (20.89)
Standardized word decoding		
WDs^c^		49.48 (29.71)

Paired sample *t* tests showed that growth in efficiency was significant between each consecutive measurement moment (all *p*’s < .001, two-tailed)

*WDc* curriculum embedded Word Decoding, *WDs* standardized Word Decoding

^a^Accuracy scores of WDc are displayed as percentages of total read words

^b^Efficiency scores of WDc are displayed as the number of correct read words per minute

^c^Efficiency score of WDs is the summation of efficiency scores of two distinct reading cards of one minute

**Table 2:** Word decoding development of the consecutive months of reading instruction

Measurement	Mean difference	SE	*t*	Effect size (Cohen’s d)
WDc1–WDc2	3.12***	.19	−16.49	.53
WDc2–WDc3	1.42 ***	.21	−6.85	.22
WDc3–WDc4	2.94***	.21	−13.95	.45
WDc4–WDc5	2.05***	.26	−7.77	.26
WDc5–WDc6	5.85***	.31	−18.88	.63

*** *p* < .001

Correlations among precursor measures, WDc scores, and WDs scores are presented in Table 3. All correlations were significant (*p*’s < .001, two-tailed). Since the WDc measures are interpreted as repeated measures of the same construct over time, the high correlations among them indicate high stability in word decoding development over time. In addition, all curriculum embedded word decoding measurements showed a high correlation with the standardized word decoding measure (e.g., *r* = .646 for WDc1–WDs). Small to medium correlations within the cognitive and linguistic precursor measures were found, indicating that the tasks measured independent abilities.

**Table 3:** Correlations between precursor measures and measures of word decoding

Measure	1	2	3	4	5	6	7	8	9	10	11	12	13
1. Phoneme isolation	–												
2. Word segmentation	.540	–											
3. G–P knowledge	.542	.572	–										
4. Rapid naming	.242	.284	.350	–									
5. Verbal STM	.414	.335	.238	.223	–								
6. Visual STM	.135	.241	.241	.222	.117	–							
7. WDc1	.354	.443	.638	.378	.202	.238	–						
8. WDc2	.342	.440	.636	.392	.197	.234	.917	–					
9. WDc3	.322	.427	.605	.384	.223	.226	.882	.923	–				
10. WDc4	.337	.418	.610	.407	.248	.219	.843	.895	.931	–			
11. WDc5	.307	.405	.585	.376	.214	.220	.794	.853	.892	.920	–		
12. WDc6	.321	.347	.555	.363	.222	.202	.710	.768	.815	.855	.898	–	
13. WDs	.287	.345	.491	.380	.224	.240	.646	.705	.731	.764	.796	.813	–

All correlations were significant with *p* < .001

*G–P* grapheme to phoneme, *STM* short-term memory, *WDc* curriculum embedded Word Decoding, *WDs* standardized Word Decoding

**Fig. 3:**
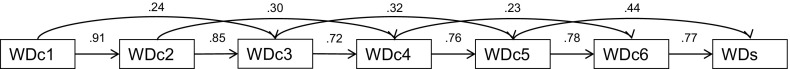
Standardized solutions of word decoding development in the first six months of reading development. *WDc* curriculum embedded Word Decoding efficiency, *WDs* standardized Word Decoding

To further answer the first research question, the efficiency scores of the consecutive measurement moments of WDc were explored in depth in a LISREL path model. In the path model, the predictive power of the consecutive WDc assessments was measured longitudinally. Standardized coefficients of the model are presented in Fig. 3. These coefficients are within-construct prediction coefficients. They indicate the average scores relative to the group mean across time; therefore, they can be interpreted as stability coefficients. Note that each WDc measurement, in addition to the influence on the consecutive measurement, systematically and independently influenced the forthcoming measurement moment thereafter. The fit of the proposed model was good, *χ* ^2^(11, N = 973) = 24.02, *p* = .02, RMSEA = .035, *χ*_*rel*_^2^ = 2.18.

**Fig. 4:**
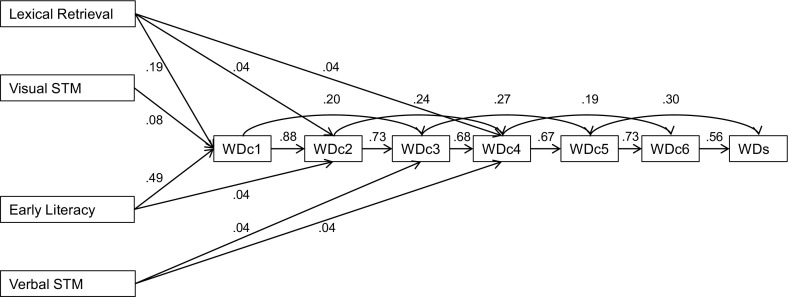
Standardized solutions of word decoding development in the first six months of reading development and precursors. *STM* short-term memory, *WDc* curriculum embedded Word Decoding efficiency, *WDs* standardized Word Decoding

The second research question addressed the predictive values of the precursor measures on early word decoding development. In order to answer this question, Early literacy, Rapid naming, and Verbal and Visual short-term memory were added to the path model (see Fig. 4). Vocabulary showed no significant independent contribution to the model after including Rapid naming, Early literacy, and short-term memory in the model; therefore, it was excluded from the model. The fit of this proposed model was good, *χ* ^2^(30, *N* = 973) = 69.87, *p* < .001, RMSEA = .037, *χ*_*rel*_^2^ = 2.33.

The models in Figs. 3 and 4 show the stability of early word decoding development during phonics instruction in the first 5 months of the first grade and the role of kindergarten precursors, as were our research questions. However, differences in schools might contribute to differences in word reading development (Chiu & McBride-Chang, 2006). For the sake of completeness, we checked whether the influence of school would alter the current path models. We did so by using fixed effect modelling, which can be interpreted as relatively stringent analysis controlling of school effects. Non-significant relations of school dummies with the word decoding measurements were preserved in these fixed effect models. Fit indices of the models that controlled for school influences were good and highly comparable with the original models, respectively *χ* ^2^(10, N = 973) = 32.54, *p* < .001, RMSEA = .049, *χ*_*rel*_^2^ = 3.25; and *χ* ^2^(30, N = 973) = 66.54, *p* < .001, RMSEA = .036, *χ*_*rel*_^2^ = 2.22. The stability coefficients were preserved in the fixed effect models, concluding that stability of word decoding development during phonics instruction remained highly stable after controlling for possible influences of school differences. Only the relation of the early literacy measure with word decoding at time 1 was stronger after controlling for school influences.

Furthermore, we checked if the stability of the model would also hold in a randomized order design. If the model results change based on the randomized order (i.e., by neglecting the fixed time factor), it further strengthens our assumption that the order of assessments matters because of its relation to the grapheme–phoneme order of instruction. Therefore, we first analysed a path model with the following random order: WDc1–WDc3–WDc5–WDc2–WDc6–WDc4–WDs. This random model had a poor fit with the data, *χ* ^2^(10) = 803.80, *p* < .001, RMSEA = .286, *χ*_*rel*_^2^ = 80.38. In addition, the stability coefficients were not convincing or even non-significant. In the randomized order model, the lags between subsequent measurements moments still contained the highest coefficients (for example the coefficient for the lag-2 of WDc5 to WDc6 is .87, while WDc5 to WDc2 is .16), showing that even in the randomized model, the model points towards an autoregressive repeated measures design with time as a fixed factor. We found similar non-fitting results with other randomized orders. These additional analyses confirmed that an autoregressive repeated measures design with time as a fixed factor was the best description of the data.

## Discussion

The present longitudinal study investigated the development of word decoding skills during incremental phonics instruction of Dutch children in the first grade. Our first research question concerned the accuracy and efficiency of early word decoding and its stability across the period of phonics instruction. In line with our hypothesis, it was found that from the very first month, mean accuracy levels reached ceiling while the mean efficiency of word decoding continued to develop after each training block, despite the incremental character of the consecutive curriculum embedded word decoding assessments. Furthermore, the autoregression in the longitudinal path model showed that the individual differences assessed by the word decoding efficiency measurements during the first 5 months of instruction had a high degree of stability over time. This means that from the very beginning of learning to read, the word decoding efficiency later in time could be predicted by levels earlier in time. Moreover, curriculum embedded word decoding efficiency highly predicted the independent standardized word decoding performance after 5 months of phonics instruction, suggesting a transfer of incrementally built-up word decoding skills to the efficient decoding of new, non-trained words.

Our second research question concerned the predictive power of precursors assessed in kindergarten on children’s early word decoding development. The current study shows that, in line with our expectations, kindergarten measures of early literacy, rapid naming, and to a lesser extent verbal and visual short-term memory predicted word decoding development during incremental phonics instruction. It can be assumed that the influence of cognitive and linguistic skills will indirectly be passed through to further steps in development from the very beginning. In accordance with our expectation, vocabulary did not add to the prediction model after including other kindergarten measures.

With respect to our first research question, the results showed that curriculum embedded word decoding accuracy reached ceiling levels already after the first month of reading instruction. From the start of phonics instruction, word decoding development is a matter of growing word decoding efficiency. These findings confirm that word reading efficiency already develops during the first months of reading development, at least in more transparent languages. This finding has been outlined, but not extensively studied, in previous research (e.g., Seymour et al., 2003; Verhoeven & Van Leeuwe, 2008; Ziegler et al., 2010). Despite the incremental character of the monthly measurements, word decoding efficiency continued to improve after each training session. This increasing efficiency might be seen as a reflection of the consolidation of the mappings of orthography with phonology and of the automation of word decoding, as claimed by the self-teaching hypothesis (Share, 2004). The results further showed that a stable path of reading development exists already from the first month of phonics instruction. Verhoeven and Van Leeuwe (2009) have previously evidenced such stability in reading development in later phases. To our knowledge, however, the current study was the first to show that stability in individual differences of reading development has already been established during, instead of after, the fundamental processes involved in mastering the alphabetic principle (see also Caravolas et al., 2013; Simmons et al., 2008; Steacy et al., 2014). The high relation of the curriculum embedded word decoding assessments with the standardized word decoding measure after 5 months indicates that the curriculum embedded measurement was an adequate way of assessing children’s performances. Furthermore, this transfer to new, nontrained words supports the claim by Share (2004) and Ehri (2005) that children should be able to read any regular word in their language as soon as they have mastered baseline word decoding skills.

With respect to our second research question, the results showed that precursors measured in kindergarten already function as predictors from the first months of word reading development. The strong predictive values of both early literacy and rapid naming skills on the development of word decoding skills are in line with the findings in previous studies on the precursors of reading development (e.g., Landerl et al., 2013; Melby-Lervåg et al., 2012; Tobia & Marzocchi, 2014; Ziegler et al., 2010), albeit these studies did not tap into the very early reading development, like the present study. Specifically, the unique predictive value of visual short-term memory that was found in the current study demonstrates that the recent findings of Van den Boer et al. (2013) and Bosse and Valdois (2009) also apply to the first months of reading development. Verbal short-term memory did not add to the prediction of curriculum embedded word decoding in the first months after controlling for early literacy and rapid naming. However, a small additional effect of verbal short-term memory after 3 months was found. This might be partly explained by the gradually increased acquisition of grapheme–phoneme correspondence rules in combination with the introduction of the more difficult digraphs (e.g., /ij/ and /oe/) and less frequently used consonants (e.g., /h/ and /w/) after 3 months. Preserving performance in later training blocks might demand extra short-term memory skills. It should be noted that this additional effect was very small. It was found that vocabulary was not independently associated with early phases of reading development after accounting for early literacy, rapid naming, and short-term memory skills. Individual differences in vocabulary might be more strongly related to later reading development, when text reading and reading comprehension skills emerge (e.g., Papadimitriou & Vlachos, 2014; Tobia & Marzocchi, 2014; Verhoeven, Van Leeuwe, & Vermeer, 2011). Additionally, the role of vocabulary in initial word reading, which has been found in some previous studies (e.g., Kirby et al., 2008; Nation & Snowling, 2004), might be more prominent in less transparent orthographies in which orthographic irregularities and several possible pronunciations for letters in a word are allowed. In more transparent orthographies, like Dutch, vocabulary often turns out to be not a statistically reliable predictor of word decoding (Caravolas et al., 2012).

A strength of the current study is its ecological validity. The instruction and assessments in the current study took place in regular classrooms, and a large representative sample in Dutch was assessed. The combination of a relatively transparent orthography and a highly systematic phonics-based reading curriculum makes it possible to study the development of initial word decoding skills without the interference of deviations and exceptions that are associated with reading in more opaque orthographies. This allows to draw more general conclusions about underlying processes in the early phases of alphabetic reading acquisition (see also Share, 2008). Of course it should be noted, however, that the current study has weaknesses too. First, although the current results hold for Dutch as a transparent orthography, the absolute time course of development of early word decoding might be different in different orthographies and countries (Vaessen et al., 2010). It is also possible that the added value of the cognitive and linguistic skills would be distributed in a different fashion across different languages, as explained in previous research (Caravolas et al., 2013; Ziegler & Goswami, 2005). The reproduction of the current design in other orthographies would be interesting, as this makes comparison between orthographies possible. Secondly, curriculum embedded assessments made it possible to reliably measure incrementally built-up word decoding development right from the beginning, in contrast to the capabilities of standardized testing. However, the use of curriculum embedded tasks instead of standardized measures has consequences for the interpretation of development. Since the content of each consecutive task was determined in parallel with the incrementally growing set of trained graphemes, growth in the word decoding efficiency cannot be interpreted as an absolute growth. It should be acknowledged that growth modelling was not appropriate in this curriculum embedded design. To place the results in a broader perspective and to see the consistency with later reading phases, curriculum embedded performances were linked to performances on a standardized word decoding task. To complete the picture of early word reading development, standardized growth models of subsequent word decoding towards consolidated alphabetic reading could be recommended in follow-up studies. Lastly, it is worth noticing that the current study has no measure of word decoding skills right before formal reading instruction started. The kindergarten assessment showed that the participating children already knew about 18 grapheme-to-phoneme correspondences before formal reading instruction started. Although actual word decoding requires more than just knowing conversion rules, it is possible, therefore, that the children were already able to read some words before instruction started. We controlled for this with an early literacy measure in kindergarten (containing grapheme–phoneme knowledge and phonemic awareness), but an actual measure of initial word decoding skill in kindergarten, containing a word decoding task of simple structured words, might have been interesting in the current study.

The current study contributes to our knowledge of early reading development. In addition to the contributions to the scientific field, the study has some practical implications. First, the results emphasize that differences between children in pre-alphabetic phases have immediate influence on the fundamental first steps of the alphabetic reading development. Therefore, full insight and fine grained documentation of cognitive and linguistic abilities of children by the end of kindergarten is relevant for Grade 1 teachers. Cognitive and linguistic information can function as markers for possible difficulties in beginning reading. Teachers should immediately anticipate the responses to instruction based on this information. Second, the current study showed the relevance of fine grained curriculum embedded monitoring of the reading processes of the children during the beginning of word decoding development, in addition to the standardized curriculum based measurements after half a year. The results of the current study emphasizes the relevance to react to small early signs of difficulties in reading development, because this might very well be related to later bigger problems in reading.

To conclude, although transitional stages from one developmental phase to another have been quite clearly described in previous studies, no clear case of mastering the alphabetic principle and the early development of word decoding and its predictors was provided yet. Our results showed that from the very beginning, children learning to read in a transparent orthography attain ceiling levels of word decoding accuracy, whereas their efficiency scores increase. Early literacy and lexical retrieval, and to a lesser extent verbal and visual short term memory, predict the first fundamental processes of learning to read. Individual differences in this early word decoding development show a high stability over time.
